# LTCC Packaged Ring Oscillator Based Sensor for Evaluation of Cell Proliferation

**DOI:** 10.3390/s18103346

**Published:** 2018-10-07

**Authors:** Joni Kilpijärvi, Niina Halonen, Maciej Sobocinski, Antti Hassinen, Bathiya Senevirathna, Kajsa Uvdal, Pamela Abshire, Elisabeth Smela, Sakari Kellokumpu, Jari Juuti, Anita Lloyd Spetz

**Affiliations:** 1 Microelectronics Research Unit, University of Oulu, P.O. Box 4500, FI-90014 Oulu, Finland.; niina.halonen@oulu.fi (N.H.); maciej.sobocinski@oulu.fi (M.S.); jajuu@ee.oulu.fi (J.J.); 2 Faculty of Biochemistry and Molecular Medicine, University of Oulu, P.O. Box 5400, FI-90014 Oulu, Finland; antti.hassinen@helsinki.fi (A.H.); sakari.kellokumpu@oulu.fi (S.K.); 3 Department of Electrical & Computer Engineering and the Institute for Systems Research, University of Maryland, College Park, MD 20742, USA; bsenevir@gmail.com (B.S.); pabshire@isr.umd.edu (P.A.); 4 Division of Molecular Surface Physics and Nanoscience, Department of Physics, Chemistry and Biology, Linköping University, SE-58183 Linköping, Sweden; kajsa@ifm.liu.se; 5 Department of Mechanical Engineering and the Institute for Systems Research, University of Maryland, College Park, MD 20742, USA; smela@umd.edu; 6 Division of Sensor and Actuator Systems, Department of Physics, Chemistry and Biology, Linköping University, SE-58183 Linköping, Sweden; spetz@ifm.liu.se

**Keywords:** capacitive sensing, cell proliferation assay, CMOS, lab-on-a-chip, low temperature co-fired ceramic, ring oscillator

## Abstract

A complementary metal-oxide-semiconductor (CMOS) chip biosensor was developed for cell viability monitoring based on an array of capacitance sensors utilizing a ring oscillator. The chip was packaged in a low temperature co-fired ceramic (LTCC) module with a flip chip bonding technique. A microcontroller operates the chip, while the whole measurement system was controlled by PC. The developed biosensor was applied for measurement of the proliferation stage of adherent cells where the sensor response depends on the ratio between healthy, viable and multiplying cells, which adhere onto the chip surface, and necrotic or apoptotic cells, which detach from the chip surface. This change in cellular adhesion caused a change in the effective permittivity in the vicinity of the sensor element, which was sensed as a change in oscillation frequency of the ring oscillator. The sensor was tested with human lung epithelial cells (BEAS-2B) during cell addition, proliferation and migration, and finally detachment induced by trypsin protease treatment. The difference in sensor response with and without cells was measured as a frequency shift in the scale of 1.1 MHz from the base frequency of 57.2 MHz. Moreover, the number of cells in the sensor vicinity was directly proportional to the frequency shift.

## 1. Introduction

Health effect evaluation of chemicals and medicines begins with cytotoxicity assays including cell cultures which are followed by animal testing. Proliferation of cells is evaluated after exposure to the assessed substance and usually includes laborious handwork required in the staining and fixing of cells for visual inspection under the microscope. Moreover, this is an end-point measurement method, which lacks real-time information on the health status of the cell population and is vulnerable to various sources of human error. In addition, marker-based cell studies require handling of potentially harmful chemicals. Animal tests are expensive, depend on specific facilities and staff, and require careful ethical considerations. Thus, there is a true demand for a cost-effective, real-time, label-free cell viability evaluation method with a high degree of automation, which highlights the need for intensive development in this technology [1].

A lab-on-a-chip (LOC) is a device which combines sampling, analyzing, and data processing on a single miniaturized platform, which makes it cost-efficient and appealing for the development of automatic biological measurements. Complementary metal-oxide-semiconductor (CMOS) technology, which was invented more than 50 years ago, has enabled more sensing, processing and computing capacity in various electrical devices. Integration of CMOS technology with the lab-on-a-chip concept forms a special variety of LOCs (Lab-on-CMOS, LOCMOS) where the actual CMOS chip is utilized in both sensing and data processing, and enables complete electronic, label-free cell assays. Several CMOS-based devices for cell sensing have been presented [2] that have exploited charge-based capacitance measurement (CBCM) [3,4], charge sharing [5,6], electric cell-substrate impedance sensing (ECIS) [7], dielectric spectroscopy [8,9,10,11], magnetic sensor [12] (needs magnetic labeling) and capacitance to frequency [5] as measurement methods, also sensors with multi-parametric measurements have been presented [13,14]. CMOS chips can be applied as biosensors for monitoring living cells or biomolecules; however, a major obstacle in their implementation is packaging of the chips for biological applications. The chips are small (<1 cm^2^) which aggravate proper shielding of electrical contacts (e.g., wire bonds) from moisture and the corrosive environment that is typical in biological systems. Due to the small size of the chip and limited possibilities in bonding, manufacturing of necessary microfluidic channels is also challenging [15]. Finally, a reusable or alternatively disposable measurement device would be preferable, but this introduces even more challenges for the packaging materials.

Low temperature co-fired ceramic (LTCC) technology has been applied for demanding sensor packages [16] and biosensor applications [17] including cell cultivation bioreactors [18,19] due to versatile material characteristics and a manufacturing process that enables diverse structures [20]. An LTCC material system solves many of the problems involved in the packaging of CMOS biosensors. Ceramic packages offer the possibility of embedded microfluidic channels inside the module. Furthermore, the cumbersome wire bonding process can be avoided with flip-chip bonding technology.

In this article, we present an LTCC packaged CMOS biosensor chip, which is utilized in measuring the proliferation of a cell population. The sensor is based on capacitive sensing with a three-stage ring oscillator that generates an oscillatory signal which is modulated by an interdigitated electrode (IDE) coupled in parallel to the second-stage. The CMOS biosensor chip consists of a 4 × 4 array of these sensor elements. When adherent cells attach and proliferate (along with other cell activities) on the IDE area the effective permittivity of the sensor surroundings decreases because cells have lower capacitance compared to the cell media and the oscillation frequency of the sensor elements increases [21]. The change in frequency is compared to an on-chip reference electrode pixel that is not exposed to the cell culture environment. To confirm that the frequency shifts are due to cell activity on the IDEs, the presence of cells was verified with microscopic imaging.

It is crucial that the electrical contact areas are not exposed to biological cell growth media, since the electric potential in the conductors in the high humidity and temperature environment would cause corrosion. LTCC technology was combined with flip-chip bonding to obtain a highly reliable and stable packaging system. Conductive and non-conductive epoxies were used to make a robust barrier between the electrical connections and the active sensor area of the chip. A similar packaging strategy has been reported with PCB and alumina packages [22,23]. In this work a successful Au-Au thermocompression flip-chip process was additionally demonstrated to bond the chip onto the LTCC substrate. This simplifies manufacturing and increases reliability. An important aspect of the packaging is biocompatibility, which was shown in earlier papers by carrying out cell viability tests [24,25]. Finally, the ring oscillator-based capacitance sensor’s suitability for sensing the presence of a viable adherent cell population was demonstrated.

## 2. Materials and Methods

### 2.1. Materials and Equipment

The sensor package in this work was made with a commercial DuPont LTCC material system consisting of LTCC tape (951) and gold (5742) or silver (6142D) paste. Tapes and vias were cut using a laser cutting system (LPKF ProtoLaser U3).

The CMOS chip was manufactured in a commercial foundry (X-FAB) using a 2-poly, 3-metal, 0.35 μm standard CMOS process. Flip-chip bonding was performed using a Finetech Electronics FINEPLACER Pico 145. The chip was attached to the LTCC substrate with Epo-Tek epoxies. Electrical connections were made with H20E-PFC silver epoxy adhesive and underfilled with EPO-TEK 377 insulating epoxy. Both epoxies were cured at 150 °C for 1 h.

The cell cultivation chamber was made from a glass or polypropylene tube (diameter 30 mm) and attached onto the LTCC module with either Casco Superfix silicone or EPO-TEK 377 epoxy. Finally, the outside of the package was passivated with Kontakt Chemie Urethane 71 spray. Oxygen plasma treatment of the sensor chip was performed with Oxford Instruments Plasmalab 80 Plus at room temperature using 30 mT chamber pressure, RF power of 100 W and O_2_ feed of 50 sccm.

The human lung epithelial cell line BEAS-2B (CRL-9609, ATCC) was used for the experiments. The cells were grown in Dulbecco’s Modified Eagle Medium (DMEM), and supplemented with 10% fetal bovine serum (FBS). Cells were washed once with phosphate-buffered saline (PBS), pH 7.4 (Thermo Fisher Scientific). Cells were grown on 10 cm culture plates in a cell incubator at 37 °C and 5% CO_2_. Cells were then detached with 0.5% trypsin-EDTA solution in PBS (pH 7.4) and the cell concentration was measured with a Countess II cell counter (Thermo Fisher Scientific). A baseline measurement for the CMOS chip was obtained with serum-free DMEM for 30 min before the addition of 10,000 cells re-suspended in the same media. After a 24 h recording with the sensor chip, cells were stained with Hoechst 33342 DNA stain and imaged using an Olympus BX-51 fluorescence microscope with a 60× LUMPlan/IR immersion objective and CellM software with appropriate filter sets. Cells were then grown in the presence of 0.5% trypsin-EDTA in serum-free DMEM for an additional 24 h to detach the cells. Staining with α-tubulin was performed on cells grown 24 to 25 h on CMOS chips treated (or not) with 0.01% poly-L-lysine (Sigma-Aldrich) and fixed with 4% paraformaldehyde in PBS (pH 7.4). Cells were then blocked with 1% BSA in PBS (pH 7.4) supplemented with 0.05% saponin. Fixed cells were stained with anti- α-tubulin antibody (Sigma Aldrich) and Alexa 488 secondary antibodies (Thermo Fisher Scientific). Cells were imaged with a Zeiss Observer. Z1 microscope equipped with an LSM 700 confocal unit, Zen2009 software (Carl Zeiss AG, Oberkochen, Germany), 63× Plan-Apo oil-immersion or 20× Plan-Apo objective and appropriate filter sets for each dye.

Cleaning of the sensor module after the experiments was done by using Tergazyme enzyme detergent (Alconox Inc.) and 70% ethanol.

### 2.2. The Ring Oscillator Based Sensor Chip

**Figure 1:**
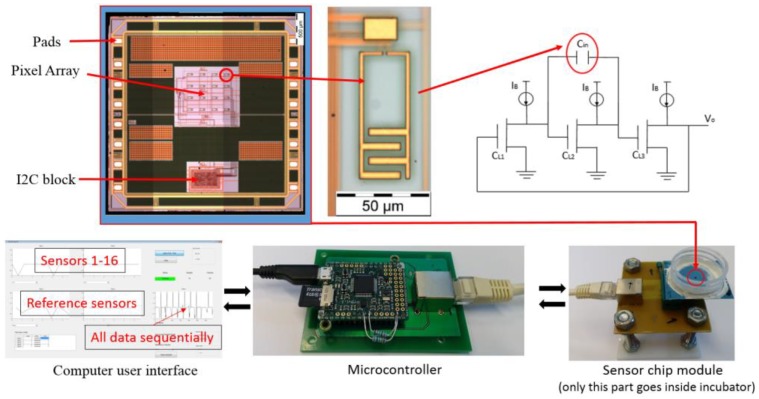
Microscope image of the sensor complementary metal-oxide-semiconductor (CMOS) chip with close-up from the capacitance sensor element and circuit diagram of the three-stage ring oscillator; below are pictures of the whole measurement system including the computer user interface is implemented with MatLAB.

The CMOS sensor chip and one of the 16 interdigitated input electrodes (inset) are shown in Figure 1. The capacitance sensors are based on capacitance-to-frequency transduction whereby an increase or decrease in sensed capacitance leads to a change in the oscillation frequency of the sensor output. The sensors are designed as three-stage ring oscillators with the input electrodes connected across one stage of the ring. This has the effect of capacitive loading of the oscillator, thus changing its frequency. The electrodes are 30 × 30 µm^2^ in size and fabricated in the CMOS process’ top metal layer. They are thus sensitive to capacitive loading caused by changes in the dielectric permittivity of the area above the surface of the chip. The output frequency of each sensor is digitized by running an on-chip 32-bit counter using the sensor signal as the clock. The counter is active for a fixed duration of time and so the final count value can be used to compute the original sensor frequency. The sensors’ transistors are separated from the chip surface by several layers of insulation and a final passivation layer, and so are shielded from the contact with cell media.

The capacitance sensors are arranged in a 4 × 4-pixel array (X and Y pitch of 196 µm and 186 µm, respectively) and all the pixels are read out sequentially. Two similar reference sensors are located outside the cell cultivation area of the chip enabling per-pixel calibration and canceling out the temperature effects. A microcontroller (MicroPython PYBv1.1) drives the chip and communicates with it using an I2C communication protocol bus. An I2C bus was chosen due to its simple protocol implementation, and because it only requires two electrical wire connections. The chip is able to sense capacitance changes in the aF range and is immune to saturation of the output signal due to the frequency sensing mechanism. A more detailed description of chip function can be found in references [26,27,28].

Figure 1 shows a schematic image of the measurement system. The measurement setup is controlled using a PC through MATLAB software, with the microcontroller connected to the PC using a USB cable. The microcontroller is connected to the sensor chip module with an RJ45 cable that helps to reduce noise on the I2C bus. The LTCC packaged sensor chip module is connected with a zero insertion force (ZIF) connector onto a PCB, so the sensor chip can be easily replaced. In actual cell measurements, only the sensor chip module is placed inside the incubator.

The MATLAB user interface shows real time plots of all 16 sensors and 2 reference sensors. Moreover, it shows sequential raw data from the chip. All sensor pixels can be monitored individually.

### 2.3. Data Post Processing

The acquired data is post-processed by using a filtering algorithm and the reference pixel data. The chip has an internal problem due to the performance of the I2C bus drivers which occasionally result in erroneous repetition of data from two sequential pixels. In such a case, this can be fixed by a cleaning algorithm that identifies and removes repeated values. The noise related to temperature changes and noise from the environment are adjusted by setting thresholds and the signal is also filtered with a 14-point percentile filter using the Origin software platform [29]. In the last step, all sensors are set to a common baseline level to compensate for manufacturing process variations, which moreover makes comparison of the sensor responses more convenient.

### 2.4. Sensing Mechanism

During their life cycle, adherent cells such as human lung fibroblasts (e.g., BEAS2B), adhere to growth surfaces, which also facilitates their movement. The cells divide mitotically and finally perish through programmed cell death (apoptosis) or necrosis. Viable, attached fibroblast cells have a flat, star-like morphology, whereas non-viable cells contract and finally detach from the growth surface [30]. Attached cells have a lower dielectric permittivity compared to the cell media which allows their detection through capacitance monitoring. When the cells are attached to the surface their permittivity influences the effective permittivity which is detected by the electrodes as a capacitance. Thus, the displacement of cell growth medium by cells causes a decrease in capacitance which is recorded as an increase of the oscillation frequency. Conversely, when cells die and thereby detach from the surface, it is detected as an increase in capacitance.

The change in frequency with/without cells is up to 1.5 MHz from baseline frequency of 57.2 MHz. This change is relative to the unloaded reference sensor. All pixels can be measured individually, which allows for the mapping of location of cells. Moreover, it is possible to estimate the number of cells on individual IDEs.

### 2.5. LTCC Package Manufacturing

**Figure 2:**
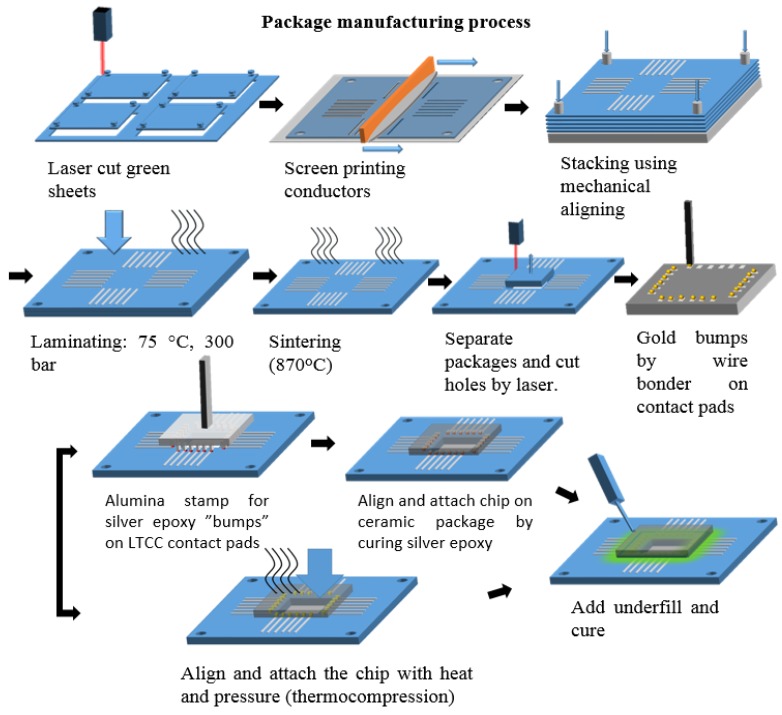
Process flow of the low temperature co-fired ceramic (LTCC) package manufacturing process using silver epoxy adhesive or thermocompression bonding (adapted from [24] in Proceedia Engineering).

**Figure 3:**
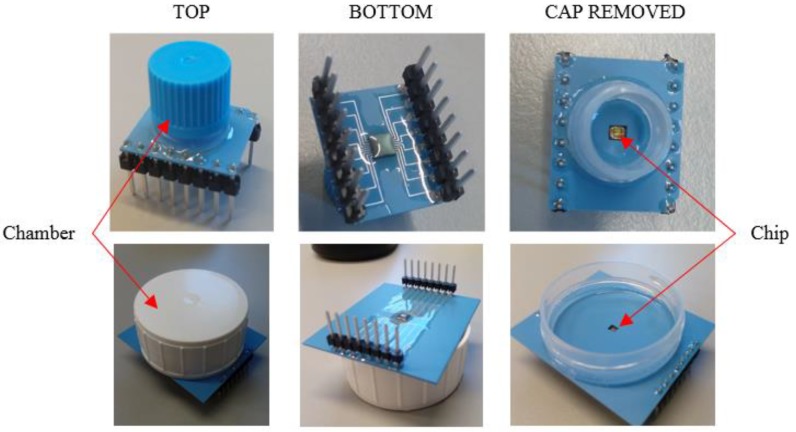
The LTCC module with sensor chip and the cell vial with two different sizes. The bigger cell vial (diameter 30 mm) enables microscope imaging with immersion lens. The footprint areas are 1.9 cm × 1.1 cm and 4.5 cm × 3.8 cm for the smaller and larger modules, respectively.

Lamination and sintering were performed according to the DuPont datasheet. An earlier presented packaging method [25] was modified to fit this application. The sensor chip was packaged onto a LTCC substrate by applying a flip-chip process. Electrical contacts were made either with conductive silver epoxy adhesive or by using an Au-Au thermocompression technique. In both cases, gold bumps were placed on the contact pads of the chip using a wire bonder (Kulicke and Soffa 4523). This facilitates the flip-chip bonding process and removes the naturally occurring non-conducting aluminum oxide layer from the pads. Epoxy underfill was used to cover electrical contacts and to seal the cavity between the chip and the LTCC substrate. The manufacturing process is shown in Figure 2. Pin headers were attached onto the LTCC package with silver epoxy. A glass or plastic tube was attached with glue leaving the chip at the bottom (see Figure 3). This served as a culture plate for the cells. The volume of the cell vial ranged from 500 µL up to 2000 µL. The largest culture plate had a diameter of 30 mm, enabling the use of an immersion objective for imaging of cells with a fluorescence microscope. The outside of the LTCC module was coated with polyurethane. The cell vial was sealed to prevent urethane from spreading to the chip or in the cell vial. The last stage was to clean the package with oxygen plasma i.e. removing all organic contaminants from the chip and to modify the chip surface, see next section, to make it more hydrophilic, thus enhancing cell attachment. A completed sensor chip package is shown in Figure 3.

### 2.6. Chip Surface Treatment

Cell adherence on top of the pristine, unpackaged CMOS sensor chip was studied and the cell adhesion was found to be insufficient. This adhesion problem on common silicon surfaces is known and reported earlier in the literature [31,32,33,34]. The top passivation layer of the chip is made of silicon nitride. Two approaches for surface treatment were tested, one with a poly-L-lysine coating and the other with oxygen plasma treatment. Poly-L-lysine coating protocol was done as provider Sigma-Aldrich instructed [35]. Oxygen plasma treatment was made using Oxford Plasmalab 80 in room temperature using vacuum of 30 mT, RF power of 100 W an O_2_ feed of 50 SCCM.

## 3. Results

### 3.1. Cell Testing

**Figure 4:**
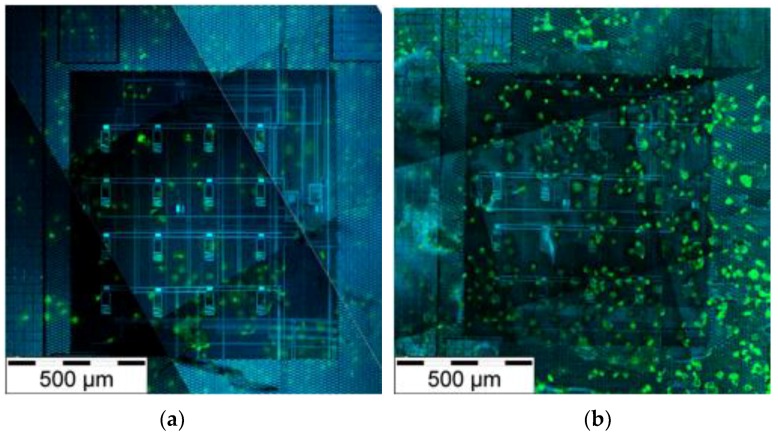
Human lung epithelial cells cultivated on the unpackaged chip. The finger electrodes were visualized with a 405 nm excitation wavelength in a confocal microscope (blue). The α-tubulin staining (green) shows the cell cytoskeleton (Alexa 488). (**a**) Pristine chip surface; (**b**) the lysine treated surface.

The chip surface was first treated with lysine (poly-L-lysine) to improve the cell adhesion. Lysine makes the surface more charged, enhancing cell attachment. Figure 4 demonstrates the difference of cell adhesion with and without lysine treatment; on a lysine-treated chip the number of cells is four-fold compared to a pristine chip. However, the lysine treatment affects the load capacitance and may interfere with the measurements by decreasing the changes in capacitance, thus the sensitivity of the sensor.

**Figure 5:**
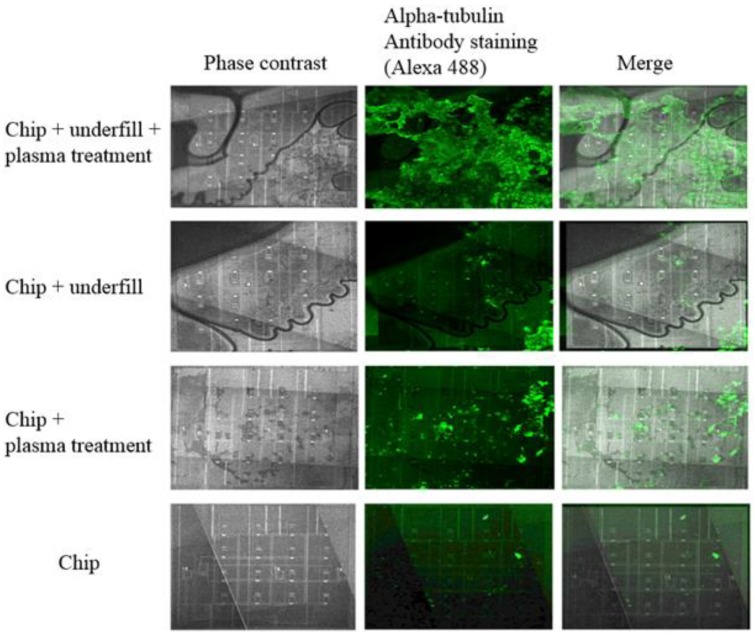
The effect of epoxy underfill and oxygen plasma cleaning on cell proliferation.

**Figure 6:**
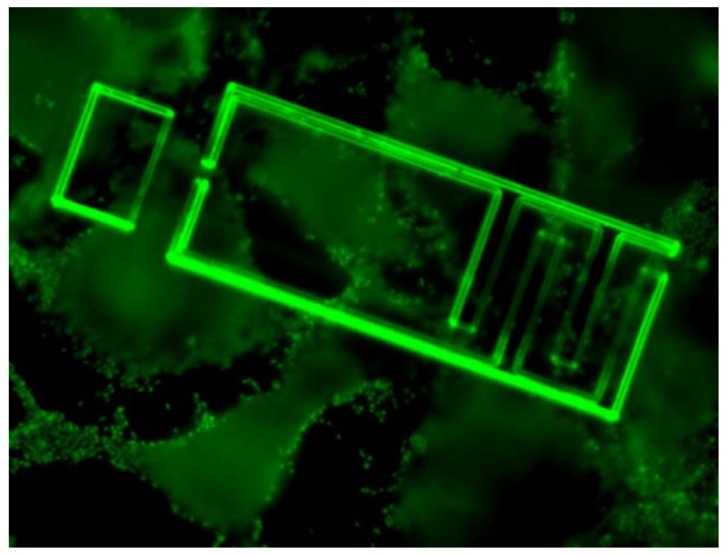
Human lung epithelial cells located on top of the sensor electrode.

The effect of oxygen plasma surface treatment on cell adhesion was also studied. The plasma treatment increased cell adhesion by transforming the chip surface to be more hydrophilic [36] and thus promoting cell adhesion (Figure 5). Figure 6 shows high magnification image of a human lung epithelial cell on sensor pixel, the cells were stained with Hoechst DNA dye and imaged with a DAPI filter set. The false coloring of 8-bit images has been adjusted from blue to green to enhance visibility.

Plasma treatment was the method chosen to enhance cell adhesion, because it does not leave excess material on the chip surface that can affect the measurements. Despite the fact that it removes contaminants and changes the surface hydrophilicity, it does not increase load capacitance.

The effect of epoxy underfill on the cell adhesion was also explored to confirm that it does not interfere with the measurement results. The cells clearly adhere on top of the epoxy layer (EPO-TEK 377), and this was enhanced by oxygen plasma treatment (Figure 5). It is clearly shown that the underfill does not affect the measurement.

### 3.2. Cell Measurements

Human lung epithelial cell adhesion and proliferation were monitored using the capacitance sensor system. After 25 h of incubation and proliferation (and other cell activities, see discussion section), 0.5% trypsin protease solution was added to detach the cells from the chip surface. In addition, as a baseline control, the data from a cell media loaded chip was recorded separately for 25 h to demonstrate that there is a clear difference in sensor response between empty and cell-loaded chips.

**Figure 7:**
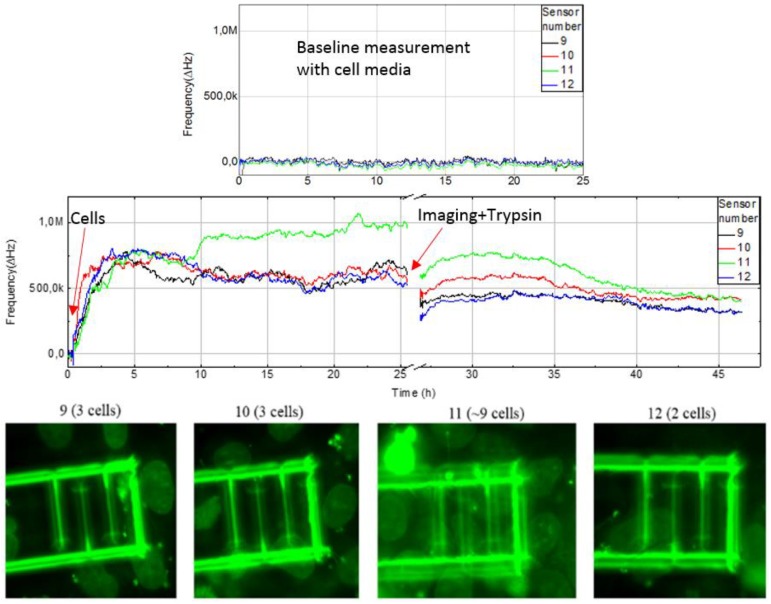
The upper graph shows the baseline measurement with only cell media and sensor response to cell addition, proliferation and detachment after trypsin addition is shown on lower graph. Signals are from the sensors 9–12 out of 16. The change in frequency is measured towards on-chip reference sensor not in contact with the cell media. Lower panel: Microscopic image of Hoechst DNA dye stained cells on pixels 9–12. The false coloring of 8-bit images has been adjusted from blue to green to enhance visibility.

**Figure 8:**
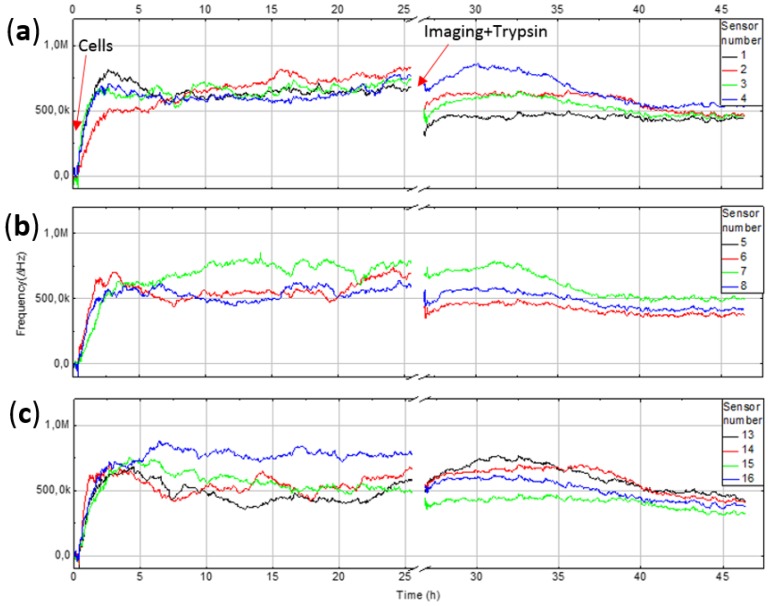
Sensor response from pixels: (**a**) 1–4; (**b**) 5–8; (**c**) 13–16.

**Figure 9:**
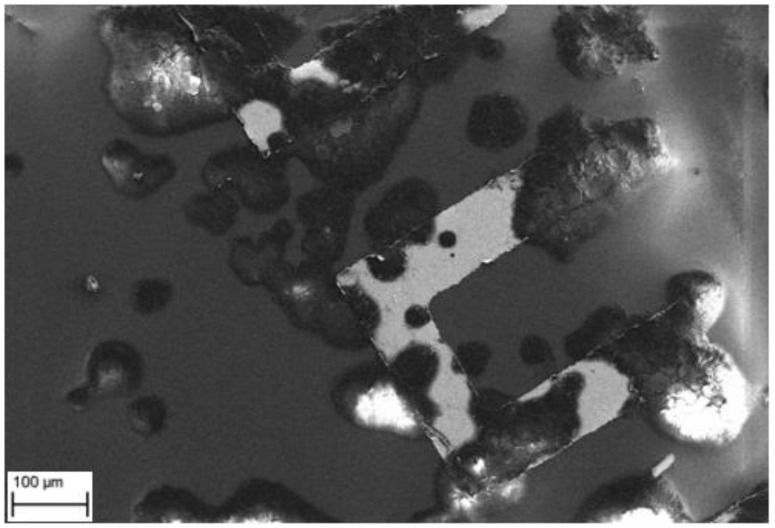
Scanning electron microscope image of dummy chip after cell detachment with trypsin treatment showing the cell protein residuals at a 300× magnification.

The sensor response from four pixels (9–12) with about 100,000 seeded cells in a cell vial (diameter 30 mm), and corresponding fluorescence microscope images are shown in Figure 7. The upper graph shows the response from measurement with only cell media, which is used as negative control. Data is plotted against the reference electrode and run through the filtering algorithm. With only cell media, the frequency change is under 50 kHz over the 25 h period. With cells the sensor shows increased frequency up to 1.5 MHz during the experiment. Sensor number 11 show a rising frequency starting at about 10 h, because there were more cells present compared to the other sensors. Frequency fluctuations are caused by dividing and moving cells. The responses from pixels 1–8 and 13–16 are shown in Figure 8. Cells were counted from the microscope images and summary of qualitative cell count and frequency shift is shown in Table 1. Sensor data was taken after 25 h just before imaging. Frequency shift is consistent with the change in number of cells on the IDEs. Pixel number 5 is not shown since it had a higher baseline due to manufacturing tolerances and therefore was removed by the filtering algorithm. The measurement was stopped while imaging was performed using a fluorescence microscope. For visualization, the cells were labeled with a Hoechst DNA stain, which binds to the nuclear DNA of the cells as small granules. The vicinity of the nuclei to the electrodes implies that the cell was at least partially covering the electrode. The coverage of the electrodes with cells was sufficient after 24 h, as on every sensor element there is at least one cell partially covering it. After microscope imaging, the measurement was restarted, and trypsin protease solution was added to induce detachment of the cells from the chip surface. As the cells were detached, the surface of the sensor electrodes is covered with cell growth medium and the sensor response drops, which indicates an increase in monitored capacitance i.e. an increase in the effective permittivity on the sensor electrodes. This is consistent with reported cell models; the cell membrane dielectric permittivity is ε_r_ = 6.2, for cytoplasm medium ε_r_ = 60, for nucleus ε_r_ = 28, for envelope ε_r_ = 52 and for the cell growth medium ε_r_ = 80, respectively [21]. These dielectric values are estimates, but the measurements indicated that the capacitive load decreased when the cells were present on the sensor electrodes. The sensor response did not go back to its original level, due to the presence of remaining protein residuals from the cells, left on the surface after the trypsin treatment. This was confirmed with a dummy chip by repeating a similar experiment and observing the chip surface with scanning electron microscope, which clearly shows cell residuals on the chip surface after trypsin treatment (Figure 9). Measurement data is also consistent with the number and coverage of cells on the chip surface, as observed with fluorescence microscope (Figure 7).

In summary, the data shows a clear response when cells are added, as they grow on the surface and finally detach after the addition of trypsin. This verifies that the capacitance (dielectric permittivity) is lower compared to the surrounding media when cells have attached onto the finger structures of the sensors.

## 4. Discussion

In the field of cytotoxicity assessment, there is a real need for new biosensors, which are label-free but above all provide real-time data about the proliferation and other cell activities of the cell cultivations. Microelectronics offers many possibilities for biosensor applications, but the bottleneck is to combine electronics with the culture conditions of adherent mammalian cells (100% humidity, +37 °C) and to find combinations of materials that are biocompatible and reliable for electronics. In the presented approach we have implemented a CMOS chip with a capacitance sensing ring oscillator packaged in an LTCC module, which provides solutions for the challenges described above. Our preliminary results are promising, but the sensor requires long-term testing in order to evaluate the stability and reproducibility of the system. More experiments are needed to fully explain why the sensor signal is not fully reversible i.e. not fully recovering to the same level as before adding the cells. For example, Figure 7 and Figure 8 show that the signal does return to the original level prior to cell addition. A contributing factor seems to be a layer of protein residuals on top of the chip after cell detachment, which insulates the electrode and lowers the net dielectric permittivity of the cell growth media. Moreover, the chip surface with cells was imaged only once giving information only at one time point. After cells attach to the surface they are not only proliferating (growth and division), but display other activities such as migration, changes in metabolism and signaling, whereby the sensor response represent the net effect of all these activities. Environmental changes such as variations in temperature during the experiment are calibrated out by using reference electrodes.

The frequency change varies between 0 and 1.1 MHz depending on the number of cells on an individual sensor pixel. When scaled to a capacitance value, this corresponds to a range of 0–1.85 fF, which is well above the measured resolution of 17.5 aF [28], corresponding to 1.92% change in the frequency with noise floor of 182 ppm.

There are residual particles left on the chip after experiments as clearly shown in Figure 9. However, it is possible to reuse the chips because of the robust packaging method, by cleaning the chip surface thoroughly. Oxygen plasma treatment could be one solution and will be tested in the future. Moreover, the epoxy underfill has a temperature limitation of 200 °C (which makes it the most temperature sensitive material of the package), and so it is possible to autoclave the whole sensor module for sterilization.

Packaging made of LTCC offers many advantages because the ease to shield the bond pads/electrical connections under the packaging material and there is no need for wire bonding. Typically wire bonds are shielded with polymers and it is difficult to avoid contamination of the active area of the chip, needing to turn the chip upside down and put for example PDMS “plug” to prevent contamination [12]. Also, the LTCC processing is matured technology capable for mass production alongside the CMOS technology.

To make full use of the advantages of LTCC packaging technology, fluidic channels can be integrated to the LTCC module making it possible to carry out flow through cell cultivation. This makes it possible to perform experiments without an incubator. However, this kind of system would also need environmental temperature control of the package. This would be possible with integrated heaters built into the package. The biggest drawback is that the fluidic channel made of for example PDMS is transparent and LTCC is opaque needing a window to enable microscopy. However, successful work has been performed to integrate a transparent window made of glass to the LTCC structure [37,38].

Another drawback is that it is difficult to achieve leveled surface between the chip and the substrate when using flip chip bonding, however this can be compensated by design of the fluidic channels so that the liquid flow is suited to the application.

## 5. Conclusions

This work has examined and produced a biosensor applied to adherent cell viability monitoring. The biosensor is based on capacitive sensing using a ring oscillator on a CMOS chip packaged in an LTCC module. Viable adherent cells attach to the sensor chip surface, while compromised cells or protease-treated cells detach from the surface. This causes a change in effective dielectric permittivity at the sensor electrodes, resulting in a change in the capacitance monitored as a shift in oscillation frequency of the ring oscillator. The sensor surface was cleaned by oxygen plasma and treated with poly-L-lysine to improve cell attachment. Oxygen plasma was observed to be an optimal surface treatment as it does not introduce residual proteins onto the chip surface. Cell adherence on the CMOS chip was tested with human lung epithelial cells (BEAS2B). The sensor showed response to cell addition, proliferation, and other cell activity, and cell detachment induced by trypsin. The sensor response was consistent with the numbers of cells and their location on the sensor elements was verified by fluorescence and confocal microscopy. The frequency difference compared to reference electrodes varied up to 1.1 MHz, depending on the number of cells on an individual sensor pixel.

## Figures and Tables

**Table 1:** Table showing summary of visible cell count on sensor versus sensor frequency change.

Sensor Number	Cell Count	Frequency Change (ΔHz)
1	5 cells, 3 on center	7.72 × 10^5^
2	4 cells on the edge	8.30 × 10^5^
3	4 cells on the edge	7.37 × 10^5^
4	4 cells on the edge	7.51 × 10^5^
5	4 cells, 2 on center	Removed (see Section 3.2)
6	4 cells on the edge	6.84 × 10^5^
7	covered in cells	7.58 × 10^5^
8	2 cells on the edge	5.66 × 10^5^
9	3 cells on the edge	5.90 × 10^5^
10	3 cells on the edge	5.37 × 10^5^
11	covered in cells	9.58 × 10^5^
12	1 cell on center, 1 on the edge	5.18 × 10^5^
13	4 cells on the edge	5.66 × 10^5^
14	4 cells, 3 on center	6.51 × 10^5^
15	2 cells on the edge	4.71 × 10^5^
16	covered in cells	7.58 × 10^5^
